# Association Between Hypnotics and Glycemic Variability Assessed by Continuous Glucose Monitoring Under Real‐Life Conditions in Patients With Type 2 Diabetes: A Cross‐Sectional Study

**DOI:** 10.1155/jdr/1748628

**Published:** 2025-12-14

**Authors:** Taichi Muramatsu, Miku Otsuka, Daisuke Yamamuro, Mikan Yamanouchi, Saaya Fujisaka, Akifumi Kushiyama, Takako Kikuchi

**Affiliations:** ^1^ Department of Pharmacotherapy, Meiji Pharmaceutical University, Kiyose, Tokyo, Japan, my-pharm.ac.jp; ^2^ Division of Diabetes and Metabolism, The Institute of Medical Science, Asahi Life Foundation, Chuo-ku, Tokyo, Japan, asahi-life.or.jp

## Abstract

**Background:**

Insomnia is common in patients with Type 2 diabetes and can negatively affect glycemic control. However, the effect of hypnotic use on glycemic variability remains unclear. Therefore, we investigated the association between hypnotic use and glycemic variability in patients with Type 2 diabetes.

**Methods:**

This cross‐sectional study enrolled patients with Type 2 diabetes who underwent continuous glucose monitoring (CGM) between June 1, 2017, and February 28, 2022. Patients were classified into six groups based on their insomnia status and hypnotic use: noninsomnia, hypnotic nonusers, benzodiazepine (BZD) users, nonbenzodiazepine (non‐BZD) users, orexin receptor antagonist (ORA) users, and melatonin receptor agonist (MRA) users. We used the standard deviation (SD) of glucose, the coefficient of variation (CV) of glucose, and the mean of daily difference (MODD) as indicators of glycemic variability. The independent association between hypnotic use and glycemic variability was assessed using a multiple linear regression model.

**Results:**

A total of 534 patients were included in the analysis (mean age: 67.7 ± 10.1 years old; mean diabetes duration: 14.5 ± 8.4 years). Thirty‐seven patients (6.9%) used hypnotics, including BZD (*n* = 13), non‐BZD (*n* = 10), ORA (*n* = 11), and MRA (*n* = 3). The SD was significantly higher in non‐BZD users (53.6 mg/dL, 95% confidence interval [CI]: 42.9–64.3) than in the noninsomnia group (40.5 mg/dL, 95% CI: 39.5–41.5). MODD was also significantly higher in non‐BZD users (50.1 mg/dL, 95% CI: 38.0–62.1) than in the noninsomnia group (35.6 mg/dL, 95% CI: 34.5–36.7). In contrast, the CV was not significantly different between non‐BZD users and the noninsomnia group. When analyzed separately for different times of the day, the nocturnal CV was significantly higher in non‐BZD users than in the noninsomnia group.

**Conclusions:**

The use of non‐BZDs was associated with within‐day and between‐day glycemic variability measured by CGM in patients with Type 2 diabetes.

## 1. Introduction

In recent decades, blood glucose management in patients with Type 2 diabetes has advanced considerably, driven by developments in pharmacotherapy and medical technology. While glycated hemoglobin (HbA1c) remains the gold standard for assessing long‐term glycemic control, it does not adequately capture glycemic variability or acute glucose fluctuations [1]. A modern approach to glycemic management emphasizes not only achieving optimal HbA1c levels but also reducing glycemic variability as much as possible [2].

Short‐term glycemic variability refers to glycemic oscillations, reflecting hypoglycemic and postprandial spikes as well as fluctuations that occur simultaneously on different days [3]. Continuous glucose monitoring (CGM) enables the precise assessment of 24‐h glycemic changes and provides data on short‐term glycemic variability [4, 5]. Short‐term glycemic variability has been associated with microvascular complications in patients with Type 2 diabetes [6–8]. Hence, appropriate management of short‐term glycemic variability is important to improve the prognosis of patients with Type 2 diabetes.

Insomnia is a sleep disorder characterized by difficulty initiating sleep, continuous sleep maintenance, or poor sleep quality [9]. The prevalence of insomnia was reported to be higher in patients with Type 2 diabetes than in those without Type 2 diabetes [10]. Furthermore, a poor sleep quality, assessed using the Pittsburgh Sleep Quality Index, was associated with increased short‐term glycemic variability in patients with Type 2 diabetes [11]. Insomnia may affect glycemic variability through multiple pathways, including impaired cerebral glucose utilization, sympathetic overactivity, disrupted circadian secretion of cortisol and growth hormone, and dysregulation of appetite‐regulating hormones [11].

Hypnotics are an effective treatment for insomnia and improve the sleep quality in patients with insomnia [12]. However, few studies have investigated the association between hypnotic use and short‐term glycemic variability measured using CGM in patients with Type 2 diabetes. Only one previous study demonstrated that suvorexant, an orexin receptor antagonist (ORA), improved short‐term glycemic variability measured by CGM in patients with Type 2 diabetes [13]. In addition to ORA, hypnotics include benzodiazepines (BZDs), nonbenzodiazepines (non‐BZDs), and melatonin receptor agonists (MRAs). These hypnotics have different mechanisms of action and characteristics, but their effects on short‐term glycemic variability in the real world remain unclear.

Therefore, the present study investigated the association between hypnotic use and short‐term glycemic variability measured using CGM in patients with Type 2 diabetes.

## 2. Methods

### 2.1. Study Design and Participants

This was a single‐center cross‐sectional study. Participants were retrospectively selected using the electronic clinical data management system. We enrolled patients with diabetes who utilized professional CGM at the Clinic of the Institute of Medical Science, Asahi Life Foundation, between June 1, 2017, and February 28, 2022. We excluded patients who met the following exclusion criteria: (i) a diagnosis of diabetes types other than Type 2 diabetes (e.g., Type 1 diabetes, pancreatic diabetes, or gestational diabetes), (ii) glutamic acid decarboxylase (GAD) antibody–positive, (iii) a diagnosis of an endocrine disorder (e.g., Cushing syndrome), (iv) a diagnosis of liver cirrhosis, (v) a history of pancreatic surgery, (vi) use of corticosteroids, (vii) a diagnosis of sleep apnea syndrome, (vii) professional CGM wearing period < 7 days, (ix) initiation of professional CGM during hospitalization, and (x) use of multiple hypnotics.

The study protocol was approved by the Ethics Committee in Meiji Pharmaceutical University (approval number: 202439), and the study was conducted in accordance with the Declaration of Helsinki.

### 2.2. Data Collection

The age at the time of professional CGM initiation was used. Sex was determined from the medical records. The body mass index (BMI) was calculated using the following equation: BMI = weight (kg)/height squared (m^2^). The duration of diabetes was calculated by subtracting the date of the diabetes diagnosis (obtained from medical records) from the date of CGM initiation. HbA1c levels were measured using an analyzer (Tosoh HLC‐723G11; Tokyo, Japan). Information on the use of hypoglycemic drugs (sulfonylureas, glinides, *α*‐glucosidase inhibitors, biguanides, thiazolidines, Dipeptidyl Peptidase‐4 [DPP‐4] inhibitors, Sodium–Glucose Transporter 2 [SGLT2] inhibitors, Glucagon‐Like Peptide‐1 [GLP‐1] receptor agonists, rapid‐acting insulins, and long‐acting insulins) was obtained from prescription history. The duration of sleep, drinking habits, and smoking status were collected using self‐reported questionnaires.

### 2.3. The Assessment of Short‐Term Glycemic Variability and Other CGM Metrics

We used the FreeStyle Libre Pro (Abbott Japan, Tokyo, Japan) to assess short‐term glycemic variability. FreeStyle Libre Pro is a professional CGM device that stores glucose levels in the interstitial fluid every 15 min for up to 14 days [14, 15]. In this study, we evaluated the standard deviation (SD) of glucose and the coefficient of variation (CV) of glucose for within‐day glycemic variability and the mean of daily difference (MODD) for between‐day glycemic variability. The SD is the rate of dispersion from the mean glucose level, and the CV is the magnitude of variability relative to the mean glucose level, calculated as follows: CV (*%*) = SD/mean glucose level × 100 [16]. The MODD is the absolute difference between two glucose values measured at the same time with a 24‐h interval [17]. In addition, we obtained the time in range (TIR), time above range (TAR), and time below range (TBR) derived from professional CGM. TIR, TAR, and TBR were defined as the percentage of time spent with glucose levels of 70–180, > 180, and < 70 mg/dL, respectively [18].

### 2.4. Classification According to Insomnia Status and Hypnotic Use

The patients were categorized into two groups based on the presence of insomnia: the noninsomnia group and the insomnia group. In addition, the insomnia group was subdivided into five groups according to their hypnotic use status: hypnotic nonusers, BZD users, non‐BZD users, ORA users, and MRA users. BZD users were defined as those who used any of the following hypnotics: triazolam, brotizolam, etizolam, rilmazafone, lormetazepam, estazolam, nitrazepam, flunitrazepam, quazepam, haloxazolam, or flurazepam. Non‐BZD users were defined as those using eszopiclone, zopiclone, or zolpidem. ORA users were defined as those who used suvorexant or lemborexant. MRA users were defined as those who had used ramelteon.

### 2.5. Statistical Analyses

Continuous variables were expressed as the mean ± SD or median (interquartile range [IQR]). Categorical variables were expressed as numbers with percentages. For each variable, the number and percentage of missing data were reported. An analysis of variance (ANOVA), Kruskal–Wallis′ test, or Fisher′s exact test was performed to compare the groups. Dunnett′s post hoc test was performed to compare the noninsomnia group with other groups. A multiple linear regression model was used as previously described [19] to evaluate the independent association of hypnotics with SD, CV, and MODD after adjusting for age, sex, duration of diabetes, HbA1c, mean glucose level, TIR, TAR, TBR, and use of rapid‐acting insulin. A complete case analysis was used to address missing data in a multiple linear regression model. Furthermore, we investigated the association between hypnotic use and short‐term glycemic variability at four separate times of the day: midnight (0:00–6:00), morning (6:00–12:00), afternoon (12:00–18:00), and evening (18:00–24:00). Statistical significance was set at *p* < 0.05.

All statistical analyses were conducted using the JMP Pro software program, Version. 18.1.0 (SAS Institute Inc., Cary, North Carolina, United States).

## 3. Results

**Figure 1:**
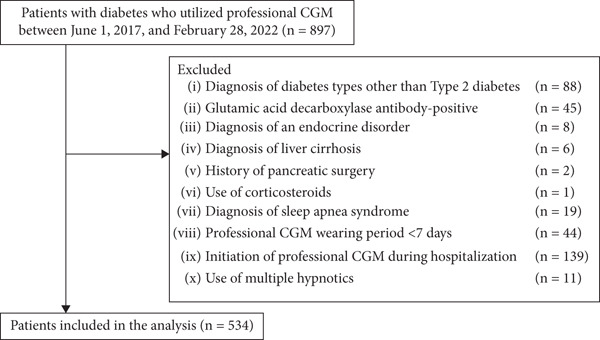
A flow diagram showing the process from enrollment to inclusion in the analysis. In the present study, 534 patients were included in the analysis. CGM, continuous glucose monitoring.

We enrolled 897 patients with diabetes who utilized professional CGM at the Clinic of The Institute of Medical Science, Asahi Life Foundation, between June 1, 2017, and February 28, 2022. A total of 363 patients were excluded from the analysis: diagnosis of diabetes types other than Type 2 diabetes (*n* = 88), GAD antibody–positive (*n* = 45), diagnosis of an endocrine disorder (*n* = 8), diagnosis of liver cirrhosis (*n* = 6), history of pancreatic surgery (*n* = 2), use of corticosteroids (*n* = 1), diagnosis of sleep apnea syndrome (*n* = 19), professional CGM wearing period < 7 days (*n* = 44), initiation of professional CGM during hospitalization (*n* = 139), and use of multiple hypnotics (*n* = 11). Ultimately, 534 patients were included in the analysis (Figure 1).

### 3.1. Patient Characteristics

The baseline characteristics of 534 patients in the analysis are shown in Table 1. The mean age of the entire cohort was 67.7 ± 10.1 years, and 419 patients (78.5%) were male. The mean duration of diabetes was 14.5 ± 8.4 years, and the mean HbA1c level was 7.3*%* ± 0.9*%*. In addition, the mean glucose level was 139.5 ± 31.2 mg/dL, the mean SD was 41.3 ± 13.3 mg/dL, the mean CV was 29.7*%* ± 7.1*%*, and the mean MODD was 36.4 ± 15.1 mg/dL. The median TIR was 77.5% (IQR: 62.5%, 86.8%), the median TAR was 14.9% (IQR: 6.9%, 26.4%), and the median TBR was 1.3% (IQR: 0.1%, 5.3%).

**Table 1:** Characteristics of 534 patients included in the analysis.

	**Total**	**Noninsomnia**	**Insomnia**					**p** **value**
			**Hypnotic nonusers**	**BZD**	**Non-BZD**	**ORA**	**MRA**	
	**n** = 534	**n** = 434	**n** = 63	**n** = 13	**n** = 10	**n** = 11	**n** = 3	
Age (years)	67.7 ± 10.1	66.8 ± 10.3	71.1 ± 8.2	75.5 ± 4.2	68.6 ± 8.3	74.5 ± 8.9	69.3 ± 3.1	0.0002
Sex (male)	419 (78.5%)	348 (80.2%)	44 (69.8%)	8 (61.5%)	7 (70.0%)	9 (81.8%)	3 (100%)	0.20
Body mass index (kg/m^2^)	24.2 ± 3.7	24.3 ± 3.8	23.7 ± 2.9	22.6 ± 3.7	23.9 ± 2.6	23.1 ± 4.6	22.0 ± 0.8	0.34
Missing data	34 (6.4%)	25 (5.8%)	5 (7.9%)	2 (15.4%)	1 (10.0%)	0 (0.0%)	1 (33.3%)	
Duration of diabetes (years)	14.5 ± 8.4	13.7 ± 8.2	18.4 ± 7.3	18.2 ± 9.6	17.6 ± 11.1	19.5 ± 10.4	11.7 ± 8.6	< 0.0001
HbA1c (%)	7.3 ± 0.9	7.2 ± 0.9	7.4 ± 0.9	7.0 ± 0.7	8.8 ± 1.7	7.0 ± 0.8	6.8 ± 0.5	< 0.0001
Missing data	1 (0.2%)	1 (0.2%)	0 (0.0%)	0 (0.0%)	0 (0.0%)	0 (0.0%)	0 (0.0%)	
CGM parameters								
Mean blood glucose (mg/dL)	139.5 ± 31.2	138.4 ± 29.8	147.1 ± 37.8	128.7 ± 21.2	166.5 ± 44.2	132.3 ± 24.3	115.7 ± 22.9	0.008
SD (mg/dL)	41.3 ± 13.3	40.7 ± 12.9	43.7 ± 13.9	42.2 ± 11.5	54.8 ± 21.9	40.3 ± 10.8	34.8 ± 15.0	—
CV (%)	29.7 ± 7.1	29.4 ± 7.2	30.0 ± 6.1	33.1 ± 8.3	32.4 ± 7.5	30.6 ± 6.0	29.7 ± 9.4	—
MODD (mg/dL)	36.4 ± 15.1	35.8 ± 14.6	38.6 ± 16.6	38.2 ± 12.7	49.3 ± 24.7	36.2 ± 12.3	30.1 ± 22.5	—
TIR (%)	77.5 (62.5, 86.8)	78.2 (63.3, 87.4)	71.6 (51.1, 83.0)	79.3 (69.1, 85.0)	53.2 (43.5, 75.0)	80.4 (65.0, 85.8)	65.0 (62.7, 94.8)	0.023
TAR (%)	14.9 (6.9, 26.4)	14.8 (6.6, 25.0)	16.2 (8.7, 35.8)	13.4 (5.1, 18.3)	44.7 (14.2, 49.0)	12.4 (7.3, 27.1)	5.0 (1.1, 15.2)	0.044
TBR (%)	1.3 (0.1, 5.3)	1.1 (0.1, 4.9)	1.7 (0.1, 8.3)	5.2 (0.3, 9.9)	3.2 (0.0, 7.4)	2.1 (0.1, 6.4)	5.5 (0.2, 17.8)	0.33
Antidiabetic drugs								
Sulfonylureas	111 (20.8%)	90 (20.7%)	14 (22.2%)	2 (15.4%)	1 (10.0%)	2 (18.2%)	2 (66.7%)	0.52
Glinides	52 (9.7%)	44 (10.1%)	5 (7.9%)	1 (7.7%)	1 (10.0%)	1 (9.1%)	0 (0.0%)	0.99
*α*‐Glucosidase inhibitors	78 (14.6%)	63 (14.5%)	10 (15.9%)	3 (23.1%)	1 (10.0%)	1 (9.1%)	0 (0.0%)	0.92
Biguanides	314 (58.8%)	261 (60.1%)	36 (57.1%)	7 (53.8%)	4 (40.0%)	6 (54.5%)	0 (0.0%)	0.29
Thiazolidines	39 (7.3%)	31 (7.1%)	5 (7.9%)	3 (23.1%)	0 (0.0%)	0 (0.0%)	0 (0.0%)	0.35
DPP‐4 inhibitors	255 (47.8%)	209 (48.2%)	29 (46.0%)	6 (46.2%)	6 (60.0%)	5 (45.5%)	0 (0.0%)	0.70
SGLT2 inhibitors	145 (27.2%)	117 (27.0%)	18 (28.6%)	4 (30.8%)	2 (20.0%)	3 (27.3%)	1 (33.3%)	0.99
GLP‐1 receptor agonists	73 (13.7%)	65 (15.0%)	4 (6.3%)	1 (7.7%)	0 (0.0%)	2 (18.2%)	1 (33.3%)	0.19
Rapid‐acting insulins	155 (29.0%)	111 (25.6%)	24 (38.1%)	8 (61.5%)	7 (70.0%)	4 (36.4%)	1 (33.3%)	0.0009
Long‐acting insulins	170 (31.8%)	129 (29.7%)	25 (39.7%)	6 (46.2%)	6 (60.0%)	3 (27.3%)	1 (33.3%)	0.15
Duration of sleep (hours)	7.2 ± 1.2	7.2 ± 1.2	7.1 ± 1.2	7.2 ± 1.3	7.0 ± 1.5	7.6 ± 1.2	—	0.89
Missing data	183 (34.3%)	134 (30.9%)	26 (41.3%)	10 (76.9%)	7 (70.0%)	4 (36.4%)	2 (66.7%)	
Drinking habits	168 (31.5%)	142 (32.7%)	15 (23.8%)	4 (30.8%)	2 (20.0%)	5 (45.5%)	0 (0.0%)	0.47
Smoking status								
Never/past/current, *n*	332/176/26	266/145/23	45/16/2	9/3/1	3/7/0	8/3/0	1/2/0	0.30
Never/past/current (%)	62.2/33.0/4.8	61.3/33.4/5.3	71.4/25.4/3.2	69.2/23.1/7.7	30.0/70.0/0.0	72.7/27.3/0.0	33.3/66.7/0.0	

*Note:* Data are presented as the mean ± standard deviation, median (interquartile range), or number (%). Comparisons among groups were performed using an analysis of variance, Kruskal–Wallis′ test, or Fisher′s exact test. *p* < 0.05 was considered statistically significant.

Abbreviations: CGM, continuous glucose monitoring; CV, coefficient of variation of glucose; DPP‐4, Dipeptidyl Peptidase‐4; GLP‐1, Glucagon‐Like Peptide‐1; HbA1c, glycated hemoglobin; MODD: mean of daily differences; SD, standard deviation of glucose; SGLT2, Sodium–Glucose Transporter 2; TAR, time above range; TBR, time below range; TIR, time in range.

The noninsomnia group (*n* = 434) was larger than the insomnia group (*n* = 100), with hypnotic nonusers (*n* = 63) comprising the majority of the insomnia group. BZD users included triazolam (*n* = 1), brotizolam (*n* = 9), etizolam (*n* = 1), rilmazafone (*n* = 1), and nitrazepam (*n* = 1). Non‐BZD users were receiving eszopiclone (*n* = 5) and zolpidem (*n* = 5). Among ORA users, all patients used suvorexant (*n* = 11). MRA users were prescribed ramelteon (*n* = 3). A comparison of baseline characteristics among the groups revealed that the age, duration of diabetes, HbA1c, mean glucose level, TIR, TAR, and use of rapid‐acting insulin differed significantly among groups.

### 3.2. Association Between Hypnotic Use and Glycemic Variability

The associations of hypnotic use with the SD, CV, and MODD after adjustment are shown in Figure 2. A total of 533 patients were included in the analysis using a multiple linear regression model, excluding one patient whose HbA1c data was missing. The SD was significantly higher in hypnotic nonusers (*p* = 0.007) and non‐BZD users (*p* = 0.0009) compared to the noninsomnia group. In contrast, no significant differences in the CV were observed between the noninsomnia group and the other groups. The MODD was also significantly higher in hypnotic nonusers (*p* = 0.016) and non‐BZD users (*p* = 0.0008) than in the noninsomnia group.

Figure 2Association between hypnotic use and the (a) SD, (b) CV, and (c) MODD. Data are presented as the adjusted means and 95% confidence intervals. We used a multiple linear regression model for adjustment. Model adjusted for the age, sex, duration of diabetes, HbA1c, mean glucose level, TIR, TAR, TBR, and use of rapid‐acting insulin. Dunnett′s test was used to compare the noninsomnia group and the other groups.  ^∗^ *p* < 0.05 and  ^∗∗^ *p* < 0.01. SD, standard deviation of glucose; CV, coefficient of variation of glucose; MODD, mean of daily difference; BZD, benzodiazepine; non‐BZD, nonbenzodiazepine; ORA, orexin receptor antagonist; MRA, melatonin receptor agonist.

**Figure:**
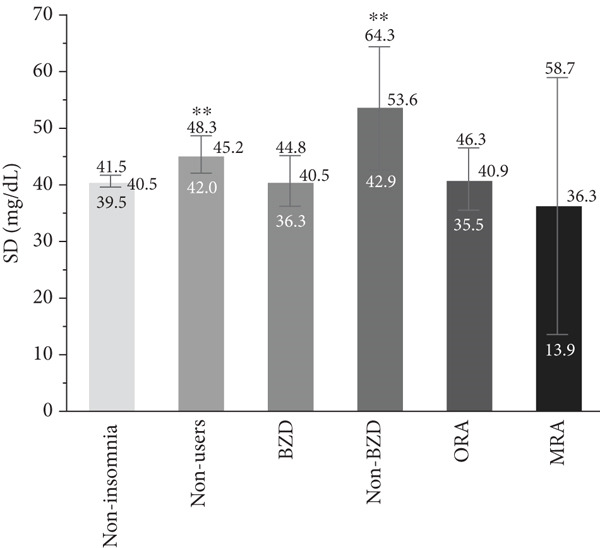
(a)

**Figure:**
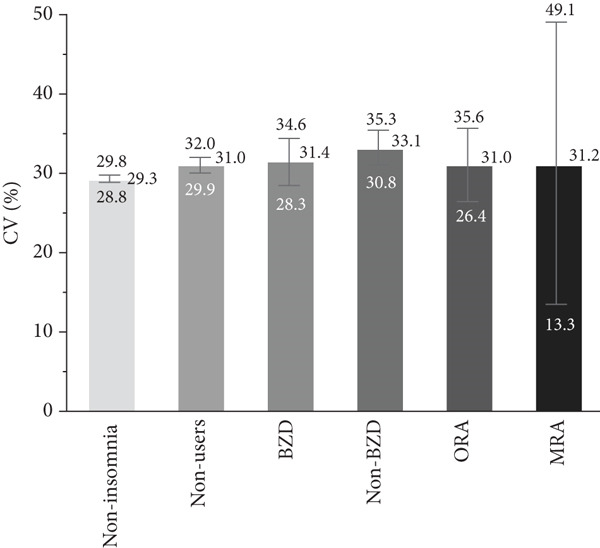
(b)

**Figure:**
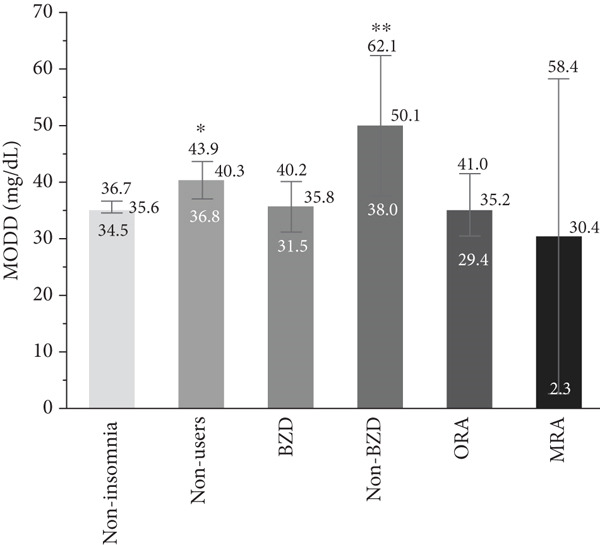
(c)

### 3.3. Association Between Hypnotic Use and Glycemic Variability at Separate Times of the Day

The association between hypnotic use and short‐term glycemic variability at separate times of the day is shown in Figure 3. The SD was significantly higher in hypnotic nonusers and non‐BZD users at all times of the day than in the noninsomnia group. Conversely, the CV was significantly higher in hypnotic nonusers at midnight (*p* = 0.033) and morning (*p* = 0.047) and in non‐BZD users at midnight (*p* = 0.002) and evening (*p* = 0.019) than in the noninsomnia group. Non‐BZD users showed a larger CV difference relative to the noninsomnia group than hypnotic nonusers. The MODD was significantly higher throughout the day in both hypnotic nonusers and non‐BZD users, except at midnight in hypnotic nonusers, than in the noninsomnia group.

Figure 3Association between hypnotic use and the (a) SD, (b) CV, and (c) MODD at separate times of the day. We divided the day into four periods: midnight (0:00–6:00), morning (6:00–12:00), afternoon (12:00–18:00), and evening (18:00–24:00). Data are presented as adjusted means and 95% confidence intervals. Dunnett′s test was used to compare the noninsomnia group and the other groups.  ^∗^ *p* < 0.05 and  ^∗∗^ *p* < 0.01. SD, standard deviation of glucose; CV, coefficient of variation of glucose; MODD, mean of daily difference; BZD, benzodiazepine; non‐BZD, non‐benzodiazepine; ORA, orexin receptor antagonist; MRA, melatonin receptor agonist.

**Figure:**
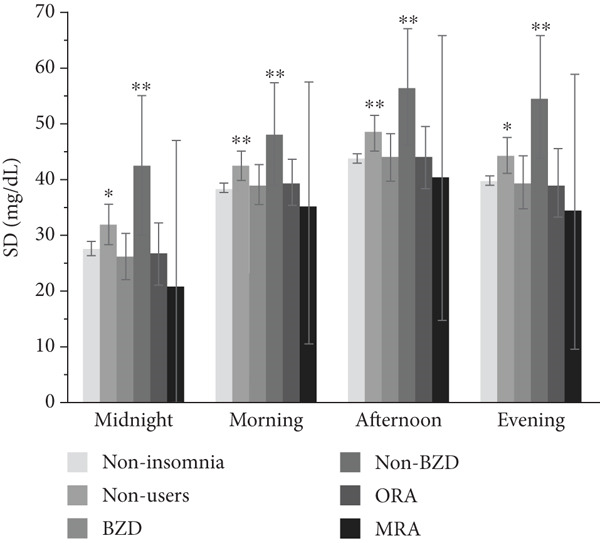
(a)

**Figure:**
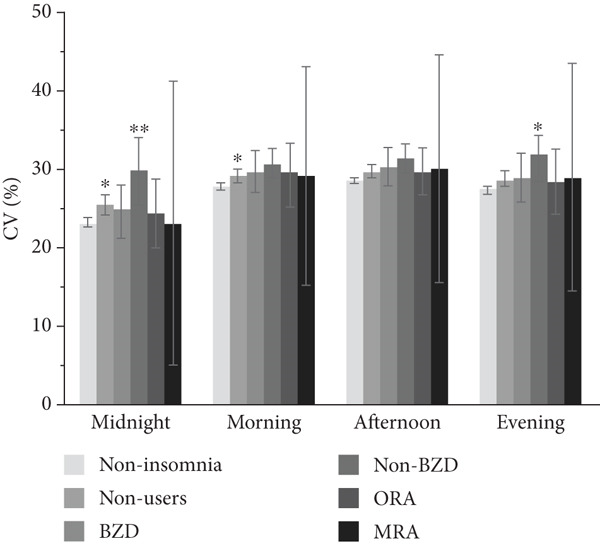
(b)

**Figure:**
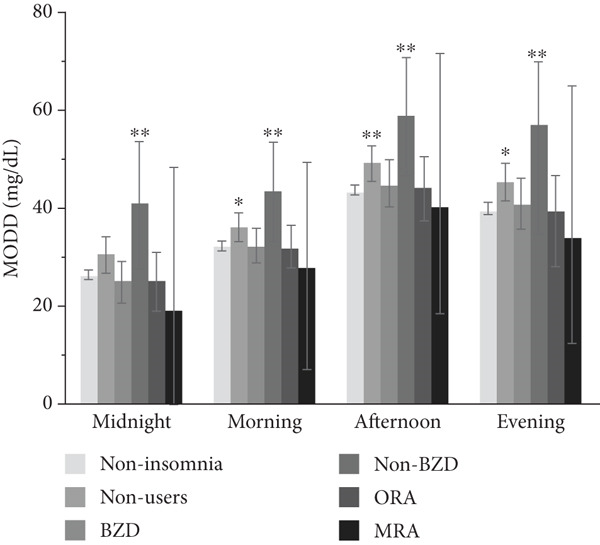
(c)

## 4. Discussion

This cross‐sectional study investigated the association between hypnotic use and short‐term glycemic variability, assessed using professional CGM, in patients with Type 2 diabetes. Notably, non‐BZD users had significantly higher SD and MODD scores than the noninsomnia group. Moreover, non‐BZD users exhibited significantly higher CVs at midnight and in the evening than the noninsomnia group.

The prevalence of insomnia was 18.7% in this study. Conversely, a previous study reported a prevalence of 39% among patients with Type 2 diabetes [20]. One possible explanation for this discrepancy is that some patients may not have reported their insomnia symptoms to their attending physicians. Alternatively, it is possible that the subjects′ symptoms may have been considered to be mild enough to be managed with advice on lifestyle modifications rather than pharmacotherapy. Consequently, physicians may not have recorded an official diagnosis of insomnia in the electronic health record, potentially leading to the inclusion of some underdiagnosed patients in the noninsomnia group. Nevertheless, patients diagnosed with insomnia in this study likely met diagnostic criteria or were deemed to require treatment. Even without pharmacological intervention, the diagnosis of insomnia was associated with a greater glycemic variability than in those without the diagnosis. This finding suggests that insomnia and its underlying factors contribute to glycemic variability.

Hypnotics were prescribed in 6.9% of the patients included in this study. This is comparable to the prescription rate (7.98%) for insomnia in Japan reported in 2021 [21], suggesting that it may reflect the actual state of insomnia treatment in Japan. In addition, the prescription rates for each hypnotic were as follows: 2.9% for BZD, 3.3% for non‐BZD, 2.7% for ORA, and 0.9% for MRA, respectively [21]. The prescription rate of each hypnotic in this study was also similar to that reported in Japan.

Recently, the American Diabetes Association/European Association for the Study of Diabetes consensus guidelines highlighted sleep as a key component of the management of Type 2 diabetes [22]. However, evidence regarding the effects of hypnotic use for insomnia on glycemic control is limited. Although a previous study has been conducted, all participants were hospitalized to minimize the impact of diet on glycemic variability [13]. The mealtime and medication schedules remained consistent throughout the hospitalization period. In contrast, we used glycemic variability in real‐life settings. Only CGM conducted in real‐life settings can indicate day‐to‐day glycemic variability, which is clearly desirable as an indication for effective and safe diabetes treatment in clinical practice. To our knowledge, this is the first study to demonstrate an association between hypnotic use and short‐term glycemic variability in real‐life settings among patients with Type 2 diabetes.

We used the SD and CV as indices for within‐day glycemic variability. The SD is significantly influenced by mean glucose levels [23], making it less appropriate than the CV when comparing patients with different mean glucose levels. In contrast, the CV is a more relevant index for the assessment of glycemic variability than the SD, as the CV is adjusted for mean glucose levels [24]. In this study, the mean glucose level was different among groups; therefore, it is reasonable to discuss the association between hypnotic use and short‐term glycemic variability based on the CV results. Although the CV was significantly higher in both hypnotic nonusers and non‐BZD users, non‐BZD users showed clinically notable elevations in nocturnal CV in this study.

The mechanism by which non‐BZD use influences glycemic variability remains unclear; thus, we can infer potential mechanisms from previous studies. Zolpidem is associated with sleep‐related eating disorder (SRED), which involves partial arousal from sleep to eat, usually occurring within the first 3 h after sleep onset [25, 26]. Patients with SRED are unaware of their behavior or behave automatically despite their awareness [27]. In the present study, non‐BZD users had significantly higher CVs at midnight and in the evening, which are times when hypnotics are taken and SRED is more likely to occur. SRED may thus contribute to increased nocturnal glycemic variability in non‐BZD users. While the mechanism underlying SRED induced by non‐BZD has not been clarified, it is suggested that disinhibition of eating behaviors via enhanced GABA_A_ receptor activity may be involved [27]. Zolpidem‐induced SRED is rare, occurring in about 1% of patients [28]. Nocturnal glucose excursions in non‐BZD users may be due to subtle changes in eating behavior that are not as severe as the phenomenon known as SRED, or SRED may not be the primary cause.

The other mechanism may involve corticotropin‐releasing hormone (CRH). CRH plays an important role in hypothalamic–pituitary–adrenocortical (HPA) axis regulation. CRH activates the secretion of adrenocorticotropic hormone (ACTH), subsequently activating the secretion of glucocorticoids [29]. The GABA_A_ receptor is expressed in the terminals of CRH neurons at the median eminence, and the GABA_A_ receptor agonist increased the intracellular calcium levels in the CRH neuron terminals in mice [30]. In addition, zolpidem immediately induces the release of ACTH after administration, which precedes the release of corticosterone in mice [31]. In contrast, BZD induces a smaller increase in plasma corticosterone levels than zolpidem [31]. These previous studies support the idea that nocturnal CV is elevated in non‐BZD users via the HPA axis.

MODD is a parameter that indicates between‐day glycemic variability, reflecting irregular dietary habits, lifestyle [32], and therapy adherence, such as insulin injection. Besides, MODD is associated with oxidative stress, a risk factor for diabetic complications [33]. Medical professionals should intervene in their dietary and medication habits, preferably with guidance, to minimize day‐to‐day glycemic variability. MODD in non‐BZD users was significantly higher than that in the noninsomnia group at all times of the day; thus, non‐BZD use may be a good marker for the risk of high day‐to‐day glycemic variability.

Glycemic variability is influenced by various clinical factors. Previous studies have shown that impaired insulin secretion is associated with worsened glycemic variability in patients with Type 2 diabetes [34, 35]. An impaired insulin secretion often necessitates insulin injections for treatment, suggesting that insulin therapy may be a marker for increased glycemic variability. The fact that a significant number of insulin treatments are administered to non‐BZD users may suggest the presence of low insulin secretion in this group. Therefore, future studies should evaluate insulin secretory capacity.

Several limitations associated with the present study warrant mention. First, the study design was cross‐sectional; therefore, causal relationships were not determined. Second, the sample size for each hypnotic user was small. Thus, careful consideration is needed to determine whether or not our results can be generalized to all patients with Type 2 diabetes. Third, there may have been unmeasured confounders owing to the retrospective design of this study. We adjusted for potential confounders that influenced glycemic variability (e.g., age, sex, duration of diabetes, and use of insulin). Finally, sleep status during the professional CGM‐wearing period was not adequately assessed. In this study, the sleep quality and insomnia severity could not be evaluated using standardized instruments such as the Pittsburgh Sleep Quality Index or the Insomnia Severity Index. Further research should consider the sleep quality and insomnia severity to more clearly elucidate the relationship between hypnotic use and glycemic variability.

## 5. Conclusion

In conclusion, the use of non‐BZD could be attributed to short‐term glycemic variability in patients with Type 2 diabetes. Careful consideration is required when prescribing hypnotics to patients with Type 2 diabetes and insomnia. Our findings provide evidence to aid the selection of hypnotics for the management of insomnia in patients with Type 2 diabetes.

## Conflicts of Interest

The authors declare no conflicts of interest.

## Funding

No funding was received for this manuscript.

## Data Availability

The data that support the findings of this study are available from the corresponding author upon reasonable request.
